# The Role of Amino Acids in Neurotransmission and Fluorescent Tools for Their Detection

**DOI:** 10.3390/ijms21176197

**Published:** 2020-08-27

**Authors:** Rochelin Dalangin, Anna Kim, Robert E. Campbell

**Affiliations:** 1 Department of Chemistry, University of Alberta, Edmonton, AB T6G 2G2, Canada; rochelin@ualberta.ca (R.D.); akim6@ualberta.ca (A.K.); 2 Department of Chemistry, Graduate School of Science, The University of Tokyo, Bunkyo City, Tokyo 113-0033, Japan

**Keywords:** amino acids, neurotransmission, fluorescence, imaging, biosensors, neurotransmitters, indicators

## Abstract

Neurotransmission between neurons, which can occur over the span of a few milliseconds, relies on the controlled release of small molecule neurotransmitters, many of which are amino acids. Fluorescence imaging provides the necessary speed to follow these events and has emerged as a powerful technique for investigating neurotransmission. In this review, we highlight some of the roles of the 20 canonical amino acids, GABA and β-alanine in neurotransmission. We also discuss available fluorescence-based probes for amino acids that have been shown to be compatible for live cell imaging, namely those based on synthetic dyes, nanostructures (quantum dots and nanotubes), and genetically encoded components. We aim to provide tool developers with information that may guide future engineering efforts and tool users with information regarding existing indicators to facilitate studies of amino acid dynamics.

## 1. Introduction

Neurons communicate to each other by the release of chemicals stored in synaptic vesicles across specialized gaps known as synapses. These chemicals diffuse across the synapse and bind to their target receptors on adjacent neurons to modulate their physiological states. While these messenger chemicals are collectively referred to as neurotransmitters, there can be confusion regarding the difference between neurotransmitters and neuromodulators. Classically, neurotransmitters are defined as molecules that meet the following criteria (adapted from Werman [1]):

- Presence of the molecule in neurons,
- Stored in synaptic vesicles and released in a Ca^2+^-dependent manner from neurons as a result of depolarization,
- Exogenous application of the molecule must elicit the same response from postsynaptic neurons as endogenously-released molecules due to binding to specific receptors, and
- The molecule must have a mechanism for its removal from the synapse.

Molecules that meet some, but not all, of these criteria can be referred to as neuromodulators. However, the term “neuromodulator” has also been used to refer to known neurotransmitters whose primary mode of action is to bind G protein-coupled receptors (GPCRs) to trigger a longer-lasting second messenger signaling cascade. To minimize confusion, we will confine the use of the term “neurotransmitter” for molecules that have met the criteria for classical neurotransmitters and refer to other molecules that can still modulate neuronal activity as “neuromodulators” from this point onward.

**Figure 1:**
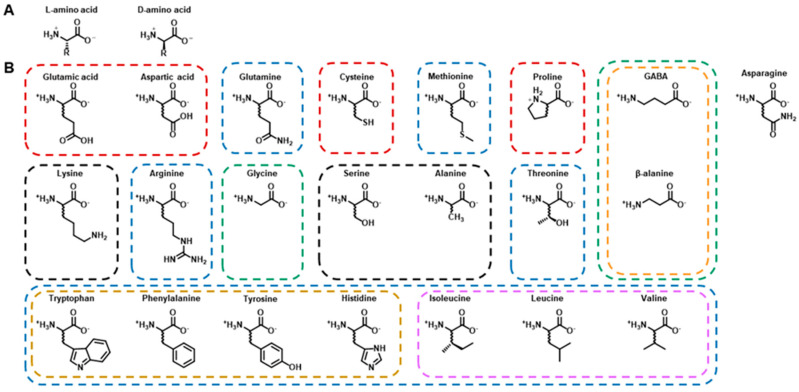
Stereochemistry of amino acids and their side chains. (**A**) Stereoisomers of amino acids are classified as D or L. The amino acids in proteins are the L stereoisomers according to the D/L system and are in the *S* configuration of the *R*/*S* system (except for cysteine which is actually in the *R* configuration due to the presence of a sulfur atom in the side chain and naming conventions). Unless stated otherwise, amino acids referred to in this review should be assumed to be the L stereoisomer. (**B**) The 22 amino acids reviewed in this paper with boxes classifying them based on their main functions. Non-proteinogenic amino acids are indicated by an orange box. The red boxes denote excitatory amino acids, while the ones in green boxes are inhibitory. Amino acids in blue boxes serve primarily as precursors for neurotransmitters, and the ones in black boxes have neuromodulatory effects. The aromatic amino acids are grouped together in a yellow box, while the branched-chain amino acids (BCAAs) are grouped in a purple box.

As a class of compounds, amino acids are most commonly recognized as the building blocks of proteins. However, strictly speaking, amino acids are defined as compounds that contain an amine group (-NH_3_^+^) and a carboxylic acid group (-COO^−^) (represented here in their physiologically most relevant ionization states; Figure 1A), and not all amino acids are proteinogenic. In addition to serving as protein building blocks, amino acids, for example, function throughout the body as key metabolites, precursors to other metabolites and lipids, and regulators of gene expression and cell signaling [2]. Within physiological systems, amino acids may also have specialized roles. In the nervous system alone, several amino acids, most famously glutamate, are known to be small molecule neurotransmitters and neuromodulators or precursors for other small molecule neurotransmitters [2]. With the prominence of several canonical amino acids in the nervous system, a review summarizing the roles of all the canonical amino acids, as well as some of the most predominant non-canonical amino acids, within the nervous system may prove to be beneficial.

In recent decades, fluorescence imaging has revolutionized our understanding of neurotransmission. Neurotransmission events can begin and conclude within milliseconds, and unlike classical methods such as microdialysis or cyclic voltammetry [3], fluorescence imaging enables the study of both single neurons and populations of neurons while maintaining high spatial and temporal resolution. Ideally, fluorescent probes (also interchangeably referred to as sensors, biosensors, reporters or indicators) will be bright, fast, specific to their target and show large intensity changes upon its detection. They should also be stable, non-toxic and be easily delivered to their target location with minimal off-target labelling. Additionally, for any analyte, sensors should be available in a palette of colors to enable simultaneous imaging of different analytes. Fluorescent probes have been synthesized using a variety of materials and strategies, each of which have their own advantages and drawbacks.

This review aims to provide a brief overview of some of the most important roles the twenty canonical amino acids, along with β-alanine and γ-aminobutyric acid, have within the nervous system. We will focus on their immediate (i.e., not their derivates’) roles in modulating neurotransmission and we will highlight the lesser known amino acids (Figure 1 and Table 1). We will also review various fluorescence-based probes for detecting endogenous amino acids in live cells and tissue. Due to the complexity and interconnectedness of neurotransmission and space limitations, this review is not meant to be exhaustive, and many relevant papers are not included.

## 2. Amino Acids

### 2.1. Glutamic Acid

Since Curtis and colleagues first reported its excitatory effects in the late 1950s, l-glutamate has been established as the main excitatory neurotransmitter in the central nervous system (CNS), with glutamatergic synapses accounting for 80 to 90% percent of the brain’s synapses and at least 60% of all the synapses in the CNS [4,5,6,7,8]. Glutamate is recycled in synapses through the glutamate–glutamine cycle [9,10]. While we aim to provide sufficient information to orient the reader for the rest of this review, due to the volume of knowledge, a thorough discussion of glutamate’s importance in neurotransmission is beyond the scope of this review and we refer readers to other reviews, such as those by Featherstone [9], Meldrum [11] and Zhou and Danbolt [12].

Glutamate concentrations in the synapse can range from less than 20 nM to 5 mM and a recent study found that glutamate concentration in isolated synaptic vesicles was approximately 700 mM [13,14,15]. Glutamate binds to three ionotropic receptors (i.e., *N*-methyl-d-aspartate (NMDA), α-amino-3-hydroxy-5-methyl-4-isoxazoleproprionic acid (AMPA), and kainate receptors), which are all channels that allow the passage of Na^+^, K^+^ and sometimes Ca^2+^. Of these, NMDA receptors uniquely function as a coincidence detector as their activation requires the binding of a co-agonist, such as glycine or d-serine, and is also voltage dependent due to a Mg^2+^ block in the pore [16,17]. Moreover, NMDA receptors conduct Ca^2+^, which acts as a secondary messenger to trigger signaling cascades. Thus, NMDA receptors are critical for synaptic plasticity and learning [18], and it has been implicated in many neurological disorders, such as addiction [19], Alzheimer’s disease [20] and others that will be mentioned in this review. Glutamate also binds to three classes of metabotropic glutamate receptors, all of which are GPCRs, that trigger different signaling cascades. Excessive activation of glutamate receptors is called excitotoxicity and leads to neuronal death and degeneration [21]. Additionally, glutamate released into the synapse can diffuse out of the synapse (“spillover”) and activate receptors outside of synapses and in other synapses [22,23,24].

### 2.2. Aspartic Acid

Aspartate is a structural homologue of glutamate, with one fewer methylene (-CH_2_) group in the sidechain. l-Aspartate was first reported to excite neurons along with l-glutamate [6,8] and is generally considered as the secondary excitatory neurotransmitter in the CNS, with some studies suggesting that aspartate and glutamate may be co-released [25,26,27]. However, unlike l-glutamate, whose role in the brain as the main excitatory neurotransmitter is well characterized and undisputed, there is still some controversy regarding the status of l-aspartate as a neurotransmitter [28,29,30].

Stimulus-dependent release of l-aspartate has been observed in different brain regions, such as the visual cortex [31], hippocampus [25,32,33] and cerebellum [34]. It was detected in the rat brain with a concentration of approximately 2.7 µmol/g wet weight, though concentrations may vary depending on the brain region (e.g., the hippocampus has 0.6 nmol/mg tissue) [35,36]. It is mostly formed from an l-aspartate transaminase-catalyzed reaction between oxaloacetate and glutamate. Storck et al. [37] demonstrated that excitatory amino acid transporter 1 (EAAT1), also known as the glutamate aspartate transporter 1 (GLAST-1), transports l-aspartate out of the extracellular space, providing a mechanism for its removal. However, the mechanism for vesicular transport remains unclear as the transporters responsible for packaging l-glutamate do not transport l-aspartate [38] and reports of a possible transporter (such as sialin) are still inconclusive [39,40]. l-Aspartate is known to be a selective agonist for NMDA receptors, but a study by Herring et al. [28] showed that l-aspartate release is insufficient for activation of NMDA receptors in the hippocampus. Furthermore, a recent profile of synaptic vesicles from cortical neurons showed no enrichment of aspartate [30]. However, a report by Richards et al. [41] found higher concentrations of aspartate than glutamate in motoneuron synapses, suggesting the possibility for physiologically relevant aspartate-evoked activation of NMDA receptors in the spinal cord. No other receptors for l-aspartate have been identified. Consequently, the significance of l-aspartate signaling remains unclear.

d-Aspartate, the enantiomer of l-aspartate, is found in the brain in significant quantities, although at concentrations ~100× lower than l-aspartate, and meets most of the criteria to be considered a classical neurotransmitter [36,42] (also reviewed by Ota et al. [43]). Found in different endocrine tissues and throughout the brain with higher levels occurring during development, d-aspartate’s roles include being an agonist for NMDA receptors, and regulating hormone release (e.g., prolactin and luteinizing hormone) and neurogenesis in developing and adult brains [44,45,46,47,48] (for a review on its neuroendocrine function, see D’Aniello et al. [49] and for a deeper discussion on its role in learning and memory, see Errico et al. [50]). Additionally, d-aspartate has been reported to activate metabotropic glutamate receptor 5 (mGluR5) [51]. The existence of specific d-aspartate receptors has also been demonstrated [42]; however, these receptors have not yet been identified. Moreover, contrary to the long-standing belief that NMDA is not endogenous in mammals, d-aspartate was also suggested to be a precursor to NMDA in rats [44]. Although serine racemase, to a degree, is able to produce d-aspartate from l-aspartate, the main synthetic pathway for d-aspartate remains an open question since reports of an aspartate racemase have been questioned [36,45,52,53,54]. However, to the best of our knowledge, like l-aspartate, the transporter responsible for loading d-aspartate into vesicles has not been identified.

### 2.3. Glutamine

Glutamine’s main role in neurotransmission is through its participation in the glutamate/GABA–glutamine cycle [9,10,54]. For a deeper discussion of the glutamate/GABA–glutamine cycle, as well as glutamine’s other roles in neurotransmission, we refer readers to the reviews by Bak et al. [10] and Albrecht et al. [55].

In glutamatergic synapses, most of the released glutamate is taken up by astrocytes, where it is converted to glutamine by glutamine synthetase. Glutamine is then exported to the extracellular space, where it is taken up by neurons and converted back into glutamate by phosphate-activated glutaminase and packaged into vesicles. Some of the synthesized glutamate may also be metabolized to aspartate. Reflecting this cycle’s importance, glutamine is found with concentrations of ~2–8 nmol/mg tissue in the brain, with the highest levels in the hippocampus and higher concentrations in the extracellular fluid (up to 1 mM) [55,56]. Glutamine metabolism is also linked to arginine/nitric oxide (NO_x_) metabolism, as glutamine synthetase both regulates, and is regulated by, NO_x_ [55,57]. Altered expression or activity of glutamine synthetase in the brain has been implicated in epilepsy [55,58], depression [59], and suicidal behavior [60], among others.

The glutamate/GABA–glutamine cycle is a key player in regulating ammonia homeostasis because one molecule of ammonia is consumed or released during the production and metabolism of glutamine, respectively. Ammonia levels must be carefully regulated as excess ammonia can trigger oxidative and nitrosative stress, which lead to increased levels of free radicals and detrimental signaling cascades [61,62]. Additionally, Albrecht and colleagues have proposed that the effects of oxidative and nitrosative stress are exacerbated by excessive glutamine synthesis, a process that consumes ammonia but is proposed to impair mitochondrial function (“the Trojan horse” hypothesis) as the excess glutamine is transported to the mitochondria as an excessive source of ammonia [61,63,64].

Evidence suggests that millimolar concentrations of glutamine can trigger currents carried by ionotropic glutamate receptors, including NMDA receptors, and induce increases in synaptic potential [65,66]. However, Luengo et al. [66] observed a decrease in field excitatory postsynaptic potential for the first 30 min upon glutamine application. The physiological relevance of this phenomenon remains unclear.

### 2.4. Cysteine and Methionine: Sulfur-Containing Amino Acids

The presence of a nucleophilic thiol group bestows cysteine and its derivatives with unique chemical properties that enable them to serve specialized functions within cells. l-Cysteine is most broadly recognized as a precursor for glutathione, the body’s main antioxidant (for more thorough discussions on the roles of glutathione in the nervous system, see the reviews by Dringen and colleagues [67,68,69]). However, despite lacking the carboxylic acid-containing side chain characteristic of excitatory neurotransmitters, l-cysteine possesses many of their characteristics. Specifically, cysteine can: (1) be released by neuron depolarization in a Ca^2+^-dependent manner, (2) activate NMDA receptors, and (3) be taken up by neurons and glia [70,71,72]. However, while l-cysteine is able to trigger synaptic activity and is a known excitotoxin, its exact mechanisms of action remain unclear (reviewed by Janàky et al. [73]). Beyond excitatory targets, Gonzáles and colleagues recently showed that l-cysteine antagonized GABA_A_ρ1 receptors [74]. l-Cysteine also acts as scavenger for acetaldehyde, the first metabolite of ethanol, reducing acetaldehyde-induced activation of the mesolimbic dopamine pathway and dampening its motivational properties indirectly [75,76,77,78]. Additionally, in the extracellular space, cysteine can be oxidized into cystine (i.e., two cysteines connected by a disulfide bond) and taken up by astrocytes through cystine/glutamate antiporter system x_c_^−^ (for a comprehensive review, see Lewerenz et al. [79]), where this extrasynaptic release of glutamate has been shown to activate extrasynaptic NMDA receptors [80]. Lastly, cysteine can be metabolized into other neuroactive compounds, such as taurine, l-cysteine sulfinic acid, l-cysteic acid and hydrogen sulfide [81,82,83,84]. Notably, taurine, an aminosulfonic acid found at a high concentration (second only to glutamate) in the brain, was shown to have an inhibitory effect on neurons by acting on GABA and glycine receptors and was consequently considered as a neurotransmitter [6,85,86,87,88,89]. More recently, however, this classification has been questioned due to the apparently lack of taurine in synaptic vesicles [30]. Regardless, a non-traditional neuromodulatory role for taurine remains a possibility with work suggesting that taurine can induce potentiation by increasing synaptic efficacy and axon excitability through intracellular accumulation [90,91].

Besides cysteine, methionine is the other sulfur-containing proteinogenic amino acid, albeit with a methylated thiol group. As an essential amino acid, methionine is transported into the CNS using the same systems used by the branched-chain and aromatic amino acids [92,93]. Methionine serves as the precursor to homocysteine, which, like cysteine, can activate glutamatergic receptors and excite neurons, even to the point of excitotoxicity through an NMDA receptor-mediated pathway [94,95,96]. In addition to activating neurons by itself, evidence also suggests that homocysteine can trigger release of other excitatory amino acids [97]. Homocysteine has been implicated in anxiety [98], alcoholism [99], Alzheimer’s disease [100] and schizophrenia [101].

### 2.5. Proline

l-Proline is a non-essential amino acid that can be synthesized from l-glutamate [102]. Hyperprolinemia, a genetic condition causing excessive levels of proline due to impaired proline metabolism, is associated with seizures, hypolocomotion, learning and other cognitive deficits, and an increased risk for schizophrenia [103,104,105]. l-Proline is a known neuromodulator in the brain and fulfills many of the criteria of a classic neurotransmitter [102,106,107,108], arguably even more so than l-aspartate, which is generally considered to be a neurotransmitter. For example, unlike l-aspartate, a vesicular transporter for l-proline, NTT4, has been identified [109]. Although a proline-specific receptor has not been identified, l-proline is a weak agonist for glycine receptors, as well as the glutamate-responsive NMDA and AMPA/kainate receptors [110]. The lower limit of the extracellular concentration of l-proline was estimated to be 10 nM [111,112]. Regardless, physiological extracellular concentrations of l-proline have been shown to modulate glutamate transmission with the ability to induce excitotoxicity [113,114,115]. Behaviorally, activation of NMDA receptors by l-proline has also been shown to mediate stress responses in chicks under acute stress by altering the stress-induced metabolism of dopamine and serotonin [116,117].

Multiple transport systems, such as the PROT transporter, have been identified for l-proline, and the specific contribution of each transport system with respect to regulating l-proline levels and their physiological importance remains unclear [118,119,120,121]. A recent study by Schulz and colleagues [122] showed that PROT^−/−^ mice lost more than 70% of l-proline uptake in brain regions where PROT is the most strongly expressed transporter such as the cortex, hippocampus, thalamus and striatum without resulting in extreme increases in extracellular l-proline concentration. However, PROT was previously shown to be more highly localized in synaptic vesicles than plasma membrane but is not considered to participate in loading l-proline into synaptic vesicles [111,112]. These vesicles were instead believed to act as a reserve pool of transporters that can then be moved to the plasma membrane to regulate l-proline uptake and neuronal activity [112]. Instead, B^0^AT2, another l-proline transporter, was proposed to be the major transporter responsible for uptake of extracellular l-proline [93]. Behaviorally, these mice showed deficits in memory extinction and locomotion, in line with the observed reductions in PROT activity and downstream effectors important in learning and memory in some regions [122,123,124]. At the same time, this study also reported that the reduction in PROT activity did not cause changes in the levels of the downstream effectors in the hippocampus, a region with one of the highest levels of PROT expression, suggesting possible compensatory mechanism in some regions.

Furthermore, l-proline has been demonstrated in rats to induce oxidative stress in the cerebral cortex, reducing the total radical-trapping antioxidant potential and increasing lipid peroxidation [125]. This proline-induced oxidative stress has been linked to proline’s inhibitory effects on both Na^+^/K^+^ pump and acetylcholinesterase activity [126,127]. Despite these advances, our understanding of proline’s role in neurotransmission and the CNS is incomplete, even more so when we consider the implications of glycine receptor activation by l-proline.

### 2.6. Asparagine

Evidence to date suggests that l-asparagine is present in the brain at low concentrations and, outside of protein synthesis, is limited to serving as a precursor l-aspartate production by asparaginases like the astrocyte-exclusive Gliap [36,128]. Asparagine can be synthesized from aspartate by asparagine synthetase, and deficiencies in this enzyme have been reported to cause brain structural abnormalities and cognitive impairments [129,130]. Asparagine is transported into the brain in competition with glutamine and histidine [92]; however, despite this competition, l-asparagine supplementation was not reported to significantly reduce glutamine levels in the brain and did not affect the levels of related neurotransmitters (i.e., glutamate, aspartate or GABA levels) in the cerebellum and medulla oblongata [131], unlike the case with BCAAs and aromatic amino acids-derived neurotransmitters (discussed below). This lack of effect is likely because asparagine can be endogenously synthesized.

### 2.7. γ-Aminobutyric Acid

γ-Aminobutyric acid (GABA) is known as the major inhibitory neurotransmitter in the brain. Although it is an amino acid, GABA is not used in proteogenesis, but functions as a signaling molecule, with the ability to induce changes in signal transduction in both presynaptic and postsynaptic neurons [132]. It is synthesized from the decarboxylation of glutamate by glutamate decarboxylase and is recycled through the GABAergic synapses in a process analogous to the glutamate–glutamine cycle [10]. GABA, upon binding to its receptors GABA_A_ and GABA**_C_**, causes chloride channels in neurons to open [132]. This can lead to depolarization in immature mammals and hyperpolarization in mature mammals [133]. Therefore, abnormal levels of GABA are commonly implicated in many psychiatric disorders, most commonly in epilepsy [134]. Other psychiatric diseases have aberrant GABA signaling. For example, late stages of Alzheimer’s disease are associated with decreased GABA levels as well as aberrant GABA_A_ receptor presence [132]. GABAergic transmission is also implicated in anxiety disorders, schizophrenia [134], Huntington’s [135] and pharmacological manipulation of GABA levels is a therapeutic strategy.

### 2.8. Lysine

**Figure 2:**
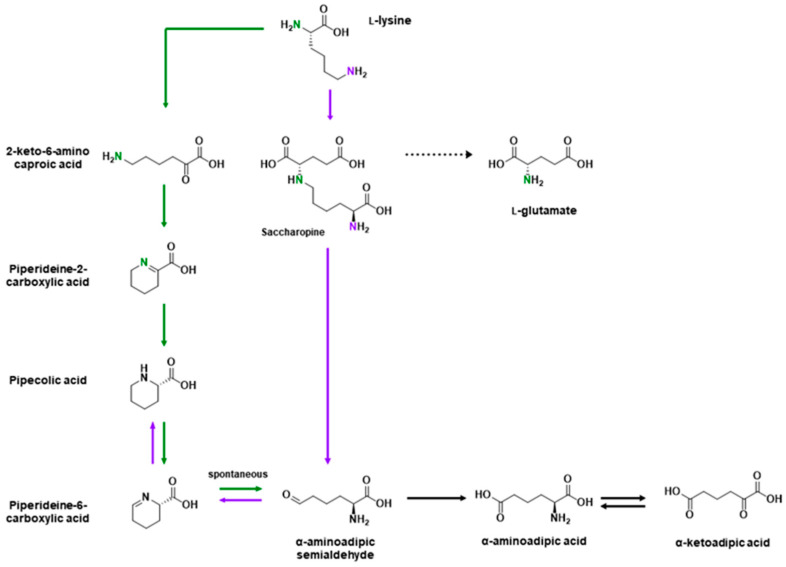
Metabolic pathways of l-lysine. l-Lysine is metabolized either through the saccharopine pathway (purple arrows) or the pipecolic acid pathway (green arrows). The two nitrogen atoms from lysine are colored in purple and green to enable atom tracing, but this distinction is lost in metabolites accessible to both pathways (i.e., pipecolic acid to α-ketoadipic acid). Adapted from Crowther et al. [142].

l-Lysine is an essential charged amino acid transported into the CNS by multiple amino acid transporters [92,118]. l-Lysine is metabolized by either the saccharopine pathway or the pipecolic acid (PA) pathway, which ultimately converge (Figure 2) [136]. While the PA pathway was long believed to be the dominant pathway in the brain [136,137], an initial report by Papes et al. [138] challenged this view and reopened the discussion. Almost a decade later, an enzyme was discovered that converts piperideine-6-carboxylic acid back to pipecolic acid, which was initially believed to be a metabolite exclusive to the PA pathway [139,140]. Subsequent work by Pena et al. [141] and Crowther et al. [142] have since shown that the saccharopine pathway is the major pathway for lysine metabolism. The distribution of lysine metabolism was of particular interest because l-lysine, through the saccharopine pathway and separate from the glutamate/GABA–glutamine cycle, is a precursor for l-glutamate, with the initial report by Papes et al. [138] estimating that approximately a third of glutamate in the CNS is from l-lysine. On the other hand, piperideine-2-carboxylic acid is an inhibitor of d-amino acid oxidase, which regulates levels of d-serine, a co-agonist of the NMDA receptor, and thus implicating lysine metabolism in schizophrenia (see review by Hallen et al. [136]).

One of the earliest discovered neuromodulatory effects of l-lysine is its effect on GABAergic transmission. In a series of works, Chang and colleagues showed that l-lysine, but not necessarily its metabolites, delayed the onset of seizures induced by pentylenetetrazol and increased seizure protection by acting through GABA_A_ receptors in a barbiturate-like manner to increase the affinity of benzodiazepines to its receptor [143,144,145]. d-Lysine was also able to delay seizure onset and confer seizure protection but with a different time course [143]. However, chronic administration of l-lysine was found to cause tolerance, with maximum protective effects peaking at 10 days of administration and decreasing when treatment time was extended to 20 days [146]. However, a clinical study by Ebrahimi and Ebrahimi [147] reported that oral administration of lysine did not reduce seizure frequency in uncontrolled epilepsy patients, suggesting that in addition to bioavailability, the type of seizure is probably relevant.

l-Lysine has also been shown to ameliorate stress-induced anxiety, likely by inhibiting serotonin (5-HT) binding to the 5-HT_4_ receptors found in the CNS and in intestines [148,149]. It was also found to be a ligand for the orphan GPRC6A receptor, which has been implicated in the endocrine system through insulin and testosterone functions [150]. l-Lysine, by itself and in conjunction with l-arginine, has also been shown to protect against ischemic insults resulting from suppression of glutamate-induced neuronal activity [151]. Recently, l-lysine was shown to affect pain-induced behavior in rats [152].

### 2.9. Arginine

l-Arginine is a semi-essential amino acid that is transported in the brain by a multitude of systems [92,118,153]. In the extracellular space of the rat brain, its resting concentration was estimated to be 17 µM [154]. Its metabolism is closely related to two other amino acids, l-citrulline and l-ornithine (reviewed thoroughly by Wiesinger [155,156]). Briefly, l-arginine can be metabolized to produce l-citrulline or l-ornithine, and it can also be recycled back from l-citrulline through the citrulline- NO_x_ cycle in neurons and glia. The main role of l-arginine in the nervous system is to serve as a precursor for NO_x_, producing citrulline as a by-product, via the activity of nitric oxide synthases. NO_x_ possesses many physiological functions, and in the brain, it plays roles in development, protection against brain injury, and learning and memory [151,157,158,159,160]. Additionally, while l-arginine’s effect on ameliorating stress-induced anxiety is likely due to NO_x_ production [148,161], evidence of l-ornithine, either directly administered or administered as l-arginine, having an ameliorating effect on stress responses suggests the possibility of a NO_x_ -independent pathway [162,163,164]. l-Arginine is also a precursor for creatine, and deficiencies in creatine synthesis have been related to different neurological conditions, such as speech impairments and movement disorders [165].

### 2.10. Glycine

Glycine is primarily synthesized from l-serine but is also metabolized to produce l-serine [166]. Glycine is the main inhibitory neurotransmitter in the spinal cord, brainstem and cerebellum, where it binds to glycine receptors (ionotropic Cl^-^ channels) when released [6,167,168,169]. A subset of synapses co-release glycine and GABA, leading to a mixture of variable cytosolic concentrations and an effective tuning of the degree of inhibition [170,171,172,173]. Released glycine is removed from the extracellular space by glycine transporters. GlyT-2 is a transporter that is mostly involved with synaptic glycine reuptake into presynaptic terminals for recycling [168,174]. GlyT-1 is involved in glycine clearance from the synapse but is also involved in the release of glycine from astrocytes in glutamatergic synapses [175]. Accordingly, it can regulate extrasynaptic glycine levels through both release and removal. Extrasynaptic GlyT-1 has an increased sensitivity to glycine [175]. It is involved in pain perception and movement, and its dysfunction has been implicated in neuropathic pain [167,176] and several startle conditions (reviewed in [177,178]). During embryonic and early postnatal development, the activation of glycine receptors is involved in cell migration and synaptogenesis with their activation causing depolarization due to the Cl^-^ gradient (reviewed by Avila et al. [179]). During development, glycine receptors tend to be expressed in the cortex though these channels would be primarily activated by taurine due to insufficient levels of glycine [179,180,181].

Glycine is also a co-agonist required for the activation of NMDA receptors [16]. At glutamatergic synapses, glycine released into the synapse is reported to spill over and activate extrasynaptic NMDA receptors preferentially (due to increased sensitivity relative to synaptic NMDA receptors) [182]. Ahmadi et al. [183] reported that, in the spinal cord, glycine released in the synapses of inhibitory interneurons can spillover out of the synapse and activate nearby NMDA receptors. This rate of glycine spill over is influenced by GlyT-1 [175,182].

### 2.11. Serine

Both enantiomers of serine are neurologically active. l-Serine acts as an important developmental and signaling molecule as well as a precursor for neuroactive molecules. l-Serine is synthesized in the brain by astrocytes using four different pathways, and deficiencies have been linked to many developmental disorders and neuropathies [184,185]. A case study has noted various developmental deficiencies such as retardation in growth, ichthyosis, polyneuropathy, and delayed puberty in one female patient [186].

A study by Buratta et al. [187] found that l-serine may be involved in the extracellular release of glutamate and aspartate through a signaling intermediate, ethanolamine. Further in vitro studies have observed that l-serine administration increased growth of the cerebellum’s Purkinje fibres and enhanced growth of dendrites in hippocampal slices [188,189]. In addition to aiding growth and the release of other amino acid neurotransmitters, l-serine also serves as a precursor to the synthesis of both glycine and d-serine, the latter of which is synthesized by serine racemase [56,166,190,191].

Although d-serine is a known neuromodulator, it does satisfy the conditions to be a neurotransmitter. Though initially reported to be a glial enzyme, serine racemase is present in significant quantities in neurons [192,193]. d-Serine competitively binds to the glycine co-agonist binding site, evoking ~90% of the glycine response [16,194]. Indeed, Papouin et al. reported that d-serine is the co-agonist for NMDA receptors [182]. Unlike most neurotransmitters, d-serine is also released by glia (reviewed by van Horn et al. [195]). Its role in NMDA modulation has implicated functions in Alzheimer’s disease and alcohol addiction, where elevated levels of d-serine were positively correlated with increased symptoms for Alzheimer’s disease on the Alzheimer’s Disease Assessment Scale, as well as to decreased dependency on alcohol use [196,197]. Comprehensive reviews by Mustafa et al. [194] and Wolosker [198] can provide detailed information on d-serine function in the brain.

### 2.12. Alanine

d-Alanine is present in brain tissues, with the highest concentration in the anterior pituitary gland (~86 nmol/g wet tissue) [36,199]. It is a known ligand for glycine receptors and can act as a co-agonist for NMDA receptors, albeit only evoking 62% of the glycine response [6,16]. Its enantiomer is also a weak agonist of NMDA receptors (evoking 12% of the glycine response) as well as glycine receptors [6,16]. d-Alanine is believed to be sourced, in part, from intestinal bacteria, with antibiotic-induced psychosis hypothesized to be caused a by a reduction in d-alanine-producing bacteria in the gut [200,201]. However, the systems involved in transporting d-amino acids through the blood–brain barrier (BBB) remain unidentified [202]. Like other d-amino acids, it is metabolized by d-amino acid oxidase [203].

Amphetamines are stimulants long known to induce hyperlocomotion through aberrant dopaminergic transmission. In 1971, Iversen et al. [204] reported that lesions on the frontal cortex, whose projections excite neurons in the caudate nucleus that inhibit motor functions, enhanced amphetamine-induced hyperlocomotion without affecting dopamine levels. This observed connection suggests that a reduction in glutamatergic transmission may be upstream of amphetamine-induced hyperlocomotion. Atsushi et al. [205] then demonstrated that d-alanine, but not l-alanine, could inhibit methamphetamine-induced hyperlocomotion, suggesting that NMDA receptor hypofunction may be responsible for the observed hyperlocomotion. Further studies identified the dopamine D_3_ receptor to be a major downstream target for these NMDA receptor-mediated locomotor effects [206].

In addition to understanding the effects of stimulants in the brain and on behavior, animal models with drug-induced manipulations of the nervous system (e.g., methamphetamine-induced hyperlocomotion or psychosis) are useful in understanding schizophrenia (reviewed by Jones et al. [207]). The initial hypothesis that schizophrenia is caused by excessive dopaminergic transmission (“dopamine hypothesis”) has since expanded to be the NMDA receptor hypofunction hypothesis, where decreased NMDA receptor function may lead to aberrant signaling, such as in dopaminergic pathways (reviewed by Olney et al. [208] and Hashimoto [209]). In line with this hypothesis, studies have demonstrated that supplementing antipsychotic drugs or d-amino acid oxidase inhibitors with d-alanine shows promise for treatment of schizophrenia [210,211].

### 2.13. Threonine

Originally probed as a possible amino acid neurotransmitter in the 1980s, threonine is a proteogenic, essential amino acid that is transported into the brain by multiple transport systems [92,118]. However, no neurotransmitter-like function has been reported, and the main non-proteinogenic role for threonine in the brain may be to a precursor for glycine [212,213]. Oral administration of threonine for those with spinal spasticity, a disorder related to aberrant peripheral nervous system (PNS) signaling, led to alleviation of spastic symptoms [214]. However, a systematic review of oral treatments for spasticity as a symptom of multiple sclerosis found that threonine administration generally did not relieve symptoms [215].

### 2.14. β-Alanine

β-Alanine is a non-proteinogenic amino acid neurotransmitter found in the CNS that is a structural intermediate of α-amino acids (e.g., alanine) and γ-amino acids (e.g., GABA). For a comprehensive review of the biochemistry of β-alanine and its role as a neurotransmitter, we refer readers to the review by Tiedje and colleagues [216].

Expanding on the evidence presented by Tiedje et al. [216] suggesting that β-alanine is a neurotransmitter, vesicular GABA transporter (VGAT) was reported to be capable of transporting β-alanine, providing a possible mechanism for β-alanine transport into vesicles [208,217]. In 2004, over forty years after the first reports of β-alanine’s inhibitory effects on neurons, Shinohara and colleagues identified β-alanine, out of over 1500 compounds, as a specific ligand for the orphan GPCR, MrgprD [218,219,220].

MrgprD belongs to the Mas-related genes, a subfamily of GPCRs expressed mostly in sensory neurons of the dorsal root ganglia. It is co-expressed with major nociceptors in a subset of small diameter neurons that exclusively target a specific layer of the epidermis, suggesting an involvement in pain modulation [218,219,221]. Early reports regarding MrgprD function found that silencing MrgprD expression reduced the sensitivity of mice to noxious mechanical stimuli by inhibiting a specific type of K^+^ current and thereby enhancing the excitability of MrgprD-expressing neurons [222,223]. MrgprD activation also opened Ca^2+^-activated chloride channels through the phospholipase C pathway [224]. Consistent with its proposed role in pain modulation, upregulated MrgprD expression caused enhanced mechanical hypersensitivity in mice models for neuropathic pain induced by chronic constriction injury [218,225]. MrgprD has been reported to play a role in the perception of noxious thermal stimuli [222,225].

MrgprD has also been implicated in histamine-independent itch mechanisms. Liu and colleagues showed that intradermal or oral β-alanine supplementation triggered an itch response in humans and confirmed with animal models that this response is mediated by MrgprD activation [226]. They also observed that β-alanine induced itch response only in a subset of MrgprD-expressing neurons, and that these neurons were also activated by heat. Taken together, these findings suggest a possible functional division between MrgprD-expressing neurons, with some neurons mediating itch and others mediating pain.

### 2.15. Aromatic Amino Acids

The aromatic amino acids consist of phenylalanine, tryptophan, tyrosine, and histidine. All but tyrosine are essential amino acids, while tyrosine is considered semi-essential because it can be synthesized by hydroxylation of phenylalanine. Therefore, tyrosine must only be consumed if insufficient phenylalanine is consumed or if the conversion of phenylalanine to tyrosine is deficient, such as in patients suffering from phenylketonuria [227]. These amino acids are transported into the CNS through the BBB, which occurs via the same transporters (and thus in competition) with other amino acids, such as the branched-chain amino acids (BCAAs) [92,118,228]. Notably, unlike the other aromatic amino acids, histidine is also transported by system N (prefers amino acids with nitrogen in the side chain), which also transports asparagine and glutamine [92]. To our knowledge, the main role of these amino acids in neurotransmission is as precursors for the synthesis of key neurotransmitters.

Tryptophan is converted into 5-HT through a two-step synthesis catalyzed first by tryptophan hydroxylase as the rate-limiting step followed by 5-HTP decarboxylase [229]. Under normal conditions, tryptophan hydroxylase is not saturated by tryptophan, thus changes to tryptophan levels in the brain, such as those caused by dietary changes, can affect the rate of 5-HT synthesis and release [229]. Indeed, the (highly variable) effects of tryptophan levels on mood (which is well known to be modulated by 5-HT) have been extensively studied (for a recent review, see Jenkins et al. [230]). Furthermore, patients with hypertryptophanemia have presented with neurological deficits such as mood swings, reduced IQs and impaired memory [231]. The kynurenine pathway, the other metabolic pathway for tryptophan, has been linked to the pipecolic acid pathway for lysine metabolism on account of shared enzymes, and this connection has been implicated in different neurological conditions (for a more thorough discussion, we refer readers to Hallen et al. [136]).

Dopamine, norepinephrine and epinephrine are sequentially synthesized from tyrosine (either taken up from diet or synthesized by phenylalanine hydroxylation) with the initial step being rate-limiting and catalyzed by tyrosine hydroxylase [229,232]. The hydroxylation of phenylalanine can also be catalyzed by tyrosine hydroxylase in the brain [233]. Acute phenylalanine and tyrosine depletion has been used to temporarily reduce dopamine synthesis with some demonstrated effects on mood and cognition [234,235,236]. Although these conditions can be controlled by a combination of dietary restrictions and/or drugs, patients suffering from hypertyrosinemia or phenylketonuria were found to have cognitive deficits relative to healthy controls [237,238].

Histidine decarboxylase converts histidine to histamine, a neurotransmitter most known for its role in regulating sleep and wakefulness but also involved in other important functions like arousal, feeding, motivation and endocrine regulation (for a comprehensive review of histamine and its roles and actions in the nervous system, we refer readers to Haas et al. [239]). Histidine decarboxylase is not saturated under normal conditions, and changes in plasma histidine levels can lead to changes in brain histidine and histamine levels [240]. However, unlike with the other aromatic amino acids, where their acute depletion is an established paradigm for manipulating neurotransmitter levels, there has been little investigation of the effects of histidine depletion on cognition [241].

### 2.16. Branched-Chain Amino Acids

Isoleucine, leucine and valine have similar biochemical properties, and are collectively referred to as the BCAAs. BCAAs are essential and must be transported into the CNS through the BBB in competition with the aromatic and other large neutral amino acids [92,118,228]. Consequently, fluctuations in BCAA levels affect the synthesis and concentrations of these aromatic amino acid-derived neurotransmitters, indirectly modulating the synthesis and release of these neurotransmitters [227,229]. For example, rats on diets supplemented with BCAA exhibited anxiety-like behaviors that can be reversed by tryptophan supplementation [242]. This relationship between BCAAs and aromatic amino acid precursors have been explored as a possible avenue for treatment of serotonin or catecholamine imbalance-related symptoms for different neurological conditions such as phenylketonuria, bipolar disorders, and anorexia, with increased BCAA intake leading to some improvements (reviewed by Fernstrom et al. in [227]). In healthy humans, there is some debate regarding the use of BCAAs to combat central fatigue, where changes in levels of serotonin and catecholamines in the CNS are believed to reduce muscle function and exercise performance, with evidence both favouring and rejecting the benefits of BCAAs (see the review by Meeusen et al. [243] for a discussion on the central fatigue hypothesis, as well as the reviews by Fernstrom et al. [227] and Newsholme et al. [244] for examples of studies analyzing the benefits of BCAAs and their possible mechanisms).

In the brain, BCAAs can also be converted into glutamate through branched-chain amino acid transaminases, replenishing the more commonly known glutamate–glutamine cycle [245]. LaNoue and colleagues [246] found that approximately 30% of de novo glutamate synthesis came from transamination of BCAAs in the retina, and the ubiquity of the branched-chain aminotransferase in the CNS suggests that BCAA transamination is a significant contributor to de novo glutamate synthesis in the rest of the CNS as well [247]. High concentrations of BCAAs, such as those found in patients with maple syrup urine disease, were found to be neurotoxic due to increased excitotoxicity and oxidative stress [248,249,250].

## 3. Fluorescence Imaging

Fluorescent probes generally consist of two components: a sensing domain that interacts with the ligand and a fluorescent reporter domain that shows a change in fluorescence intensity upon ligand binding. In this review, fluorescent sensors will first be categorized by their component scaffolds’ type (i.e., synthetic dye based, genetically encoded single fluorescent protein (FP) based, quantum dots (QDs) based, nanotubes based, or hybrids), consisting of single or non-interacting fluorophores, with the last section focusing on Förster Resonance Energy Transfer (FRET)-based sensors, which require transfers of energy between two fluorophores, using these different scaffolds. Additionally, although there is an array of fluorescent sensors available for visualizing amino acids, especially for synthetic dye-based sensors, we will confine our review to sensors that have been demonstrated in live cells with limited toxicity.

For the sake of this review, we will be summarizing the past work in the area and stating the various sensors that have been reported. However, it is important to consider that not all of the reported sensors provide the same degree of performance and some only possess small signal changes that may render them impractical for many applications. In addition, the quality of the reported data is also highly variable, with some sensor characterization data seeming to be of questionable quality [251,252,253,254,255,256,257,258,259,260,261,262,263]. More specifically, in the course of preparing this review, we found that the data (such as the spectra, affinity titrations, or specificity tests) for some reported sensors did not appear to be internally consistent within a single publication. We caution that researchers using these sensors perform their own validation and run parallel experiments with a non-responsive control construct.

### 3.1. Synthetic Dye-Based Indicators (Excluding FRET-Based Sensors)

Synthetic dye-based indicators can be employed for the detection of amino acids (Table 2). Generally speaking, synthetic dye-based indicators can provide a convenient method for imaging the concentration of their respective analytes, often showing large responses due to their turn on/off nature and fast response kinetics, though many designs involve an irreversible reaction to detect their target, and are not applicable to imaging dynamic reversible changes. Unlike simpler ions, (such as metal cations, non-metal anions and small polyatomic ions), which have more readily available synthetic sensors using a range of different recognition moieties (often referred to in the literature as “synthetic receptors”) [264,265,266,267], amino acids have a common backbone and different (yet typically quite flexible) side chains, which complicates efforts to design synthetic receptors for amino acids with high specificity. This difficulty is because synthetic receptors require precise spatial organization of small organic and inorganic molecule building blocks, which are not significantly larger than amino acids, to form complexes with their targets. Thus, because of the limited availability of synthetic amino acid receptors, many of the available synthetic dye-based sensors require a reaction to detect their targets, though significant strides have been made in recent years in designing synthetic amino acid receptors [268]. In addition, synthetic dye-based indicators may show poor photostability and be toxic to cells [269,270]. For a review of synthetic dyes and a comparison with quantum dots, we refer readers to the review by Resch-Genger et al. [270]. Table 1.

Many previously reported efforts have focused on the synthesis of dyes for cysteine detection, with at least 24 synthetic dye-based reported to function in detecting cysteine in the past five years (Table 2) [257,258,259,260,261,262,263,271,272,273,274,275,276,277,278,279,280,281,282,283,284,285,286,287,288]. Of these, several can be targeted to the mitochondria [262,282,285,286], the Golgi apparatus [259,276], the endoplasmic reticulum [274,275] and the lysosome [277]. Five act as non-specific sensors, detecting cysteine and other molecules that contain thiol groups or cysteine metabolites, with some using different wavelengths to distinguish between the different ligands [258,271,273,280,283]. These synthetic sensors all require irreversible reactions to detect cysteine, negatively affecting kinetics and requiring at least 5 min to an hour for maximum fluorescence [257,259,260,262,271,272,273,274,275,276,277,278,279,280,281,282,283,284,285,286,288].

Outside of these cysteine- and thiol-sensitive dyes, three synthetic sensors, one based on coumarin and the other two based on naphthalimide were published for the detection of histidine in cells (Table 2) [289,290,291]. The first, CAQA, was reported to be specific but retained a significant response to cysteine and other thiols present in cells; cells were treated with a thiol scavenger to eliminate any interfering thiols [289]. The other two can reversibly detect histidine, show emission wavelengths at similar ranges (~530 nm), but have different upper limits of detection and localization patterns. Next, NCH-Cu^2+^, is reversible and shows good specific response between zero and 5 µM as well as possible sublocalization to lysosomes [291]. Lastly, NPC shows a linear response up to 16 µM and has been demonstrated to be applicable in HeLa cells and *Caenorhabditis elegans* [290]. However, NPC does show modest responses (<1-fold) to other amino acids.

Finally, two synthetic sensors have been reported for aspartate. A green Cu^2+^-dependent aspartate-sensing synthetic reporter, 8MPS, was shown to detect exogenously added aspartate in live MCF-7 cells and *C. elegans* but retained a significant response to other amino acids (Table 2) [292]. The second, N,N-SP-BPY, showed the largest fluorescent change towards aspartate and glutamate, but also responds to other amino acids, especially cysteine [256]. The 8MPS and the histidine sensors require Cu ^2+^ ions to quench the sensor’s fluorescence while the presence of histidine or aspartate rescues the fluorescence. They suffer from the same limitations, as similar levels of fluorescence may be observed with the presence of both Cu^2+^ ions and the ligand of interest or with neither present. Therefore, although they can image the presence of the amino acids, measurement of real-time flux of each amino acid may be difficult. To our knowledge, these are the current extant amino acid sensors capable of being used in live cell imaging.

### 3.2. Genetically Encoded Single FP-Based Indicators

Genetically encoded indicators are a popular class of indicators for neuronal imaging due to their ease of delivery (i.e., plasmid transfection or packaged into viruses) and the specificity of their targeting (e.g., expression in different organelles or in a specific subset of cells). These indicators consist of a ligand binding protein, usually a periplasmic binding protein (PBP) or a GPCR, as the sensing domain and a fluorescent protein as the reporter domain such that binding of the ligand by the sensing domain induces a change in the chromophore’s environment, causing a change in fluorescent intensity. Unlike synthetic dye-based amino acid indicators, which often required the synthesis of a recognition moiety, genetically encoded indicators often capitalize on naturally occurring proteins that have evolved to have specificity and affinity for binding their target. The first single fluorescent protein-based indicator for an amino acid was iGluSnFR, a glutamate indicator that used a glutamate/aspartate binding protein from *Escherichia coli* and green fluorescent protein (GFP) (Table 2) [293]. Though it showed a greater response to l-glutamate, it retained a smaller response to l-aspartate with comparable affinity. Since then, a functionally brighter variant as well as different chromatic variants, ranging from blue to red, have also been reported [294,295]. Further engineering of iGluSnFR also led to different variants with different kinetics, sensitivities, or affinities [294,296,297].

Single fluorescent protein-based indicators have also been developed for GABA and histidine (Table 2). The iGABASnFR series utilized a GABA-binding protein from *Pseudomonas fluorescens* and had different variants possessing a range of affinities and dynamic ranges [298]. iGABASnFR was also shown to have low affinity for glycine, alanine and histidine. Its applications in mice models and zebra fish for detecting concentration changes in GABA were also demonstrated. Notably, however, its use for imaging GABA events longer than 1 s in duration may be limited as it undergoes a second fluorescence change after 1 s.

On the other hand, the yellow histidine indicator, FHisJ, used the HisJ binding protein from *E. coli* and showed a 520% increase in the fluorescence excitation ratio at 420 and 485 nm (R_485/420_) when histidine is added [299]. FHisJ has a high affinity for histidine but does show a three-fold increase in R_485/420_ in response to 100 mM l-arginine. The authors also expressed FHisJ in the cytosol and the mitochondrial matrix of HeLa cells, where they used FHisJ to estimate the histidine concentration (~159 and 77 µM, respectively) and to study histidine transport into cells.

### 3.3. Nanostructures (Excluding FRET-Based Sensors)

#### 3.3.1. Quantum Dots

QDs are semi-conductor nanoparticles with optical and chemical properties that are influenced by their size. They are attractive for biological investigations because of their brightness, narrow (and tunable) emission profiles (which facilitates multiplex imaging), high photochemical and thermal stability, resistance to photobleaching and long fluorescence lifetimes [270]. However, QDs are limited by possible toxicity depending on their composition (especially cadmium-based QDs [300]), challenges associated with their delivery for intracellular applications, and their tendency to “blink” (intermittent periods of no observable emission) [270]. We refer readers to the review by Resch-Genger [270] for a discussion of their properties as well as a thorough comparison of quantum dots against synthetic dyes.

A red sensor using copper indium sulfide (CuInS_2_)-based quantum dots functionalized with tyrosine was reported as a sensor for cysteine, glutathione, histidine and threonine (Table 2) [301]. For this sensor, the addition of copper (II) ions quenches the fluorescence, which can then be restored by the addition of the ligands. However, this sensor appears to also respond to aspartate and tryptophan and its use has not been demonstrated in living cells. Another sensor, using bright yellow carbon dots functionalized with *o*-phenylenediamine and GABA, has also been reported to detect histidine specifically (Table 2) [302]; however, similar to the CuInS_2_-based sensor [301], this sensor requires the addition of a fluoroqinolone to first quench the fluorescence before recovery with histidine [302]. Testing of these carbon dots in human hepatoma cells showed good intracellular uptake with minimal cytotoxicity, suggesting that they may be used to image intracellular histidine dynamics in living cells.

#### 3.3.2. Carbon Nanotubes

Carbon nanotubes are semi-conducting hollow tubes of graphene that are categorized based on their thickness as either single-walled carbon nanotubes (SWCNTs), consisting of one layer of graphite (and are thus an allotrope of carbon), or multiwalled carbon nanotubes (MWCNTs). Although their lengths may vary, carbon nanotubes have a diameter ranging from one to several nm. Carbon nanotubes are a promising scaffold for building biosensors because of their unique physical and chemical properties (for thorough discussions on carbon nanotubes, we refer readers to the reviews by Liu et al. [303], Kruss et al. [304], and Yang et al. [305]). From a fluorescence imaging perspective, SWCNTs are of particular interest because they possess tunable near-infrared emission profiles [306]. The emission profile of these carbon nanotubes is preferable to that of most other sensors using different building blocks as light in this region allows for greater penetration [307,308]. Carbon nanotubes can be functionalized by coating them with biomolecules, forming a “corona”, to tweak their properties, such as in order to confer specificity towards a target analyte or increase solubility. This strategy has been employed, using DNA or RNA for the corona, to engineer SWCNTs for the detection of catecholamines, a class of key neurotransmitters [309,310,311]. Although carbon nanotube-based fluorescent sensors, to our knowledge, do not yet exist for amino acids, the successful development of sensors for catecholamines, which are derived from amino acids, bode well for the development of carbon nanotube-based fluorescent sensors for amino acids.

### 3.4. Hybrid Strategies (Excluding FRET-Based Sensors)

Hybrid sensors for amino acids incorporate a genetically encoded component for sensing the amino acid and a synthetic flurophore as the reporter. This approach combines the advantages of proteins’ specificity for their ligands with the brightness of synthetic dyes but requires the delivery of a dye in the system. Additionally, hybrid sensors are, by design, modular since the synthetic dyes can be replaced; however, in reality, replacing the dye may affect the sensor’s dynamic range.

The first hybrid sensor that was demonstrated in cells was for glutamate, dubbed glutamate (**E**) **O**ptical **S**ensor (EOS), and utilized the S1S2 glutamate binding domain of the GluR2 subunit of AMPA receptors with a cysteine mutation engineered for attaching an environmentally-sensitive fluorophore (Table 2) [312]. The first-generation EOS showed a modest response (∆F/F_min_ = 0.20) on the cell surface but was sufficient for mapping synaptically-release glutamate in hippocampal cultures. Two improved EOS variants with improved dynamic ranges were then shown to be used in slices and in vivo [313]. However, tethering of all these EOS variants required the unspecific labelling of EOS and cells with biotin by chemical reagents. In 2014, a high throughput development system was used to engineer enhanced EOS (eEOS) which showed a ∆F/F_min_ of 5 (comparable to iGluSnFR [259]) on the surface of cultured neurons [314]. In this work, the unspecific biotinylation of the cell surface was avoided by conjugating eEOS to biotinylated BoNT/C-Hc, a domain of a neurotoxin that binds to gangliosides on neuronal surfaces. Recently, a hybrid glutamate sensor, named Fl-GluBP has been reported [297]. Fl-GluBP utilizes the same binding protein as iGluSnFR, exhibits a ∆F/F_min_ of 1.9, and retains a significant response to glutamine (∆F/F_min_ = 1.5). Although this sensor remains untested in cells, its similarities to iGluSnFR suggest that Fl-GluBP should also be applicable in cells. Lastly, a hybrid GABA sensor using the same binding domain as iGABASnFR showed a ∆F/F_min_ of ~0.7 (Table 2) [298].

### 3.5. FRET-Based Sensors

FRET-based sensors require a donor fluorophore that, upon excitation, transfers its energy to an acceptor fluorophore without emission of a photon. The efficiency of this transfer, known as the FRET efficiency, is dependent on the distance and orientation of the fluorophores as well as the spectral overlap between the emission spectrum of the donor and the absorption spectrum of the acceptor. Ligand binding induces a change in the distance and orientation of the fluorophores, causing a change in the ratios of fluorescence intensities of both donor and acceptor fluorophores. The presence of two fluorophores is both advantageous, since their 1:1 normalizes any changes caused by differences in expression and allows for quantification, and disadvantageous, since the two fluorophores consume more spectral bandwidth and limit the possibilities for multicolor imaging.

Most FRET-based sensors for amino acids are genetically encoded sensors that utilize PBPs from bacteria as the ligand binding domain with cyan variants of GFP as the donor and yellow variants as the acceptor. Genetically encoded FRET sensors for cysteine [251], glutamate [23,315], glycine [316], histidine [317], isoleucine [252], lysine [254,318], leucine [253], methionine [255], glutamine [319], arginine [154,317,320], and tryptophan [321] have been reported (Table 2). Of these, the first arginine sensor [320] is unique as it uses the glutamine binding protein from *E. coli* as its recognition motif, while the glycine indicator, GlyFS [316], utilized a binding domain that originally bound GABA, proline and alanine and was engineered to bind glycine. The latest arginine sensor utilized an arginine-binding protein identified from ancestral protein reconstruction [154]. Additionally, FRET sensors that recognize multiple ligands have also been reported, such as one for lysine and arginine [317], aspartate and glutamate [317], and BCAAs [317,322]. FRET sensors that use l-(7-hydroxycoumarin-4-yl)ethylglycine, an unnatural fluorescent amino acid, for glutamine and methionine have also been reported [323,324]. Beyond genetically encoded sensors, two irreversible FRET-based synthetic probes selective for cysteine with applications in mammalian cells are also recently available (Table 2) [325,326].

SNAP tag-based **i**ndicator proteins with a fluorescent **i**ntramolecular **t**ether (Snifits), are hybrid FRET-based sensors for glutamate and GABA (Table 2) [327,328]. Snifits consist of a receptor protein fused to both SNAP and CLIP tags, which are two orthogonal tags that can be used for the attachment of FRET-capable fluorophores, that is also tethered to a competitive antagonist. Displacement of the competitive antagonist induces a change in FRET efficiency, which can then be quantified. The glutamate sensor, called Snifit-iGluR5 for the glutamate receptor used as the binding protein, showed a decrease in FRET efficiency upon glutamate binding (∆R/R_min_ = 0.9 for the purified sensor and 0.6 on the surface of HEK293T cells) [327]. On the other hand, GABA-Snifit is based on the metabotropic GABA_B_ receptor with a decrease in FRET efficiency for a ∆R/R_min_ of 0.8, while a variant with a GB1/2 chimera instead of the GABA_B_ receptor, which could bind ligands but not interact with G proteins, showed ∆R/R_min_ of 0.4 with reduced affinity [328].

## 4. Conclusions

Amino acids have specific, but interconnected, roles for proper neurotransmission (Table 1). Beyond their role in protein synthesis, many of the proteinogenic amino acids possess neuromodulatory effects while others act as essential precursors to neurotransmitters without which deficiencies in neurotransmission will result. Additionally, due to the shared nature of the amino acid transport systems, perturbations in the levels of some essential amino acids may affect others. Despite the significant strides made in understanding neurotransmission in recent decades, there is much more that needs to be clarified, especially with respect to the roles amino acids have in neurotransmission. Indeed, several amino acids, including some d-amino acids, are known to have neurotransmitter-like effects, yet key mechanistic questions about their release—and their neurological relevance—remain unanswered.

Fluorescence imaging is a powerful technique that has the potential to answer many of these unresolved questions and advance our understanding of neurotransmission. However, its potential is handicapped by the limited availability and performance of sensors for amino acids. Out of the 22 amino acids reviewed here, sensors whose use has been demonstrated in living cells have only been reported for 14 amino acids (Table 2). Our survey of available fluorescent probes for amino acids revealed that most synthetic dye-based sensors are for cysteine and other biological thiols, taking advantage of the unique nucleophilicity of thiols. Similarly, despite the advantages they offer, there is a limited number of QD-based sensors. None are carbon nanotube-based, but given the platform’s infancy, we believe that carbon nanotube-based sensors for amino acids would be forthcoming. On the other hand, sensors which utilized amino acid-binding proteins have been reported for 13 amino acids, suggesting that strategies that incorporate an amino acid-binding protein as the recognition motif might provide the fastest route for sensors. Although existing sensors with genetically encoded recognition motifs have generally relied on known periplasmic binding proteins, recent advancements in utilizing GPCRs as a scaffold [329] and protein engineering for engineering specificity for new ligands [330] should facilitate the engineering of new and better biosensors for amino acids.

Ultimately, however, the most effective strategy would be through the collaborative efforts of tool developers, using a combination of materials and strategies, and researchers who intend to use these tools for their investigations. Open feedback loops between developers and users will maximize the impact of tool development efforts and lead to further advancements in our understanding of neurotransmission.

## Figures and Tables

**Table 1:** Amino acids and their roles in neurotransmission. See main text for complete references.

Amino Acid	Excitatory or Inhibitory	Neurotransmitter, Neuromodulator, or Precursor	Receptor	Function
Glutamic acid (Glu)	Excitatory	Neurotransmitter	Ionotropic (AMPA, NMDA, and kainate) metabotropic glutamate receptors	Main excitatory neurotransmitter in CNS [9,11,12]. Can spill over for extrasynaptic activation [22,23,24]. Excesses can cause excitotoxicity [21].
Aspartic acid (Asp)	Excitatory	Neuromodulator, neurotransmitter	NMDA and mGluR5 (d-asp only) [51]	l-Asp—neuromodulator (proposed neurotransmitter) [28,29]. d-Asp—neuromodulator (proposed neurotransmitter) [42,43]; involved in hormone release, neurogenesis, learning and memory [49,50].
Glutamine (Gln)	N/A	Precursor	Ionotropic glutamate receptors (but requires millimolar concentrations) [65,66]	Generation of glutamate, GABA, and aspartate [10,55]. Involved in regulating ammonia homeostasis [63,64]. Unclear physiological relevance of glutamine-induced activation of ionotropic glutamate receptors.
Cysteine (Cys)	Excitatory	Neurotransmitter, precursor	NMDA [71,73]	Physiological relevance of NMDAR activation is unclear. Excitotoxin—unknown mechanism [73]. Precursor to glutathione, taurine, l-cysteine sulfuric acid, l-cysteic acid and hydrogen sulfide [81,82,83,84].
Methionine (Met)	N/A	Precursor	N/A	Precursor to homocysteine, which is an excitatory neuromodulator that binds to NMDA receptors [94,95,96].
Proline (Pro)	Excitatory	Neuromodulator	Glycine, NMDA, and AMPA/Kainate [110]	Excess leads to hyperprolinemia (seizures, hyperlocomotion, learning and other cognitive deficits) [103,104,105]. Stress response [116,117].
Asparagine (Asn)	N/A	Precursor	N/A	Precursor to aspartate [36,128]. Deficiencies in synthesis leads to structural abnormalities in brain and cognitive deficits [129,130].
GABA	Inhibitory (adult); excitatory (developing)	Neurotransmitter	Ionotropic (GABA_A_) and metabotropic (GABA_B_)	Major inhibitory neurotransmitter in the brain. Co-released with glycine in some synapses [170,171,172,173].
Lysine (Lys)	Inhibitory	Neuromodulator, precursor	GABA_A_ and GPRC6_A_ [150]	Precursor for l-glutamate [138]. Modulator of GABAergic transmission [143,144,145,146]. Indirect regulation of d-serine [136]. Stress response and pain [148,149].
Arginine (Arg)	N/A	Precursor	N/A	Precursor to NO_x_ species and creatine [155,156]. Reduces stress-induced anxiety [148,161,162,163,164].
Glycine (Gly)	Inhibitory	Neurotransmitter	Glycine receptors and NMDA	Main inhibitory neurotransmitter in the spinal cord [6,167,168,169]. Co-released with GABA in some synapses [170,171,172,173]. Co-agonist of (extrasynaptic) NMDA receptors [16,182]. Involved in cell migration and synaptogenesis [179].
Serine (Ser)	Both	Precursor, neurotransmitter	NMDA and glycine (d-ser)	l-Ser—precursor to glycine and d-serine [56,166,190,191]; facilitate release of glutamate and aspartate [187]. d-Ser—co-agonist for glycine and NMDA receptors [16,182]; involved in Alzheimer’s disease and alcohol addiction [196,197].
Alanine (Ala)	Both	Neuromodulator	Glycine and NMDA	d-Ala—weaker agonist for glycine receptors and co-agonist for NMDA receptors [6,16].
Threonine (Thr)	N/A	Precursor	N/A	Precursor to glycine [212,213].
β*-*alanine (β-Ala)	Inhibitory	Neurotransmitter, precursor	MrgprD [218], NMDA, GABA_A/C_, and glycine [216]	Rate-limiting precursor to carnosine. Pain modulation [219,222,225]. Histamine-independent itch mechanisms [226].
Aromatic amino acids (phenylalanine (Phe), tryptophan (Trp), tyrosine (Tyr) and histidine (His))	N/A	Precursors	N/A	Precursor to catecholamines, serotonin and histamine [229,232,239].
BCAAs (isoleucine (Ile), leucine (Leu) and valine (Val))	N/A	Precursor	N/A	Competes with aromatic amino acid transport, indirectly modulating synthesis of catecholamines, serotonin and histamine [227,242]. Precursor for glutamate [246,247].

**Table 2:** Fluorescent indicators for amino acids.

Ligand	Type	Name	Multiple Variants	Color	∆F/F_min_ or ∆R/R_min_	Response In Vitro ^a^	Ref.
Glu	Synthetic	N,N-SP-BPY	No	Green	∆F/F_min_	~8.8 ^b,c^	[256]
	Genetically encoded (GE)	iGluSnFR		Green	∆F/F_min_	4.5	[293]
		iGlu*_f_* andiGlu_u_	Yes			3.0	[296]
		sf-iGluSnFR		Blue to green		4.5	[294]
		R-iGluSnFR		Green and red		3.9	[295]
		iGlu_l,m,h_	Yes	Green		2.4	[297]
	FRET (GE)	FLIPE	Yes	Cyan/yellow	∆R/R_min_	0.27	[315]
		SuperGluSnFR	No			0.44	[23]
		FLIP-cpGltI210				0.31	[317]
	Hybrid	EOS	No	Green	∆F/F_min_	0.37	[312]
		EOS-K716A and EOS-L401C	Yes			0.48	[313]
		eEOS	No			24	[314]
		Fl-GluBP	No			1.9	[297]
	Hybrid FRET	Snifit-iGluR5	No	Green/far red	∆R/R_min_	0.93	[327]
Asp	Synthetic	8MPS	No	Green	∆F/F_min_	~30 ^c^	[292]
		N,N-SP-BPY				~8.8 ^b,c^	[256]
	FRET (GE)	FLIP-cpGltI210	No	Cyan/yellow	∆R/R_min_	0.31	[317]
Gln	FRET (GE)	FLIP-cpGlnH183	No	Cyan/yellow	∆R/R_min_	0.13	[317]
		FLIPQ	Yes			0.26	[319]
		EGFP_-10_-GlnBP-N138CouA	No	Blue/green		0.89	[323]
Cys	Synthetic	Probe 1		Blue	∆F/F_min_	66	[271]
		Probe 1		Blue		~120 ^c^	[272]
		NCQ		Blue/green		~4.7, 3 ^c^	[273]
		Nap-Cys			∆R/R_min_	22	[274]
		TCS		Cyan	∆F/F_min_	25 ^b^	[257]
		Probe 1		Green		130 ^b^	[258]
		GT-Cys			∆F/F_min_	110 ^b^	[259]
		NPCC				13	[275]
		Gol-Cys				20	[276]
		Ly-1				8.8	[277]
		CyP				~33 ^c^	[278]
		Compound 1				~9 ^c^	[279]
		BDY-NBD		Green/NIR		~7400, 9.8 ^c,d,e^	[280]
		hCy-A		Green/red	∆R/R_min_	~8 ^c^	[281]
		PYR			∆R/R_min_	163	[282]
		XCN		Red	∆F/F_min_	1081	[283]
		P-Cy				3	[260]
		DCIP				~5 ^b,c^	[261]
		CyA		NIR		~6.5 ^c^	[284]
		Cy-S-diOMe				250	[285]
		NFL1				~20 ^c^	[286]
		DDNA				31 ^f^	[287]
		CP-NIR				40	[288]
		Mito-CP				12 ^b^	[262]
		DP-NIR				7.5 ^b^	[263]
	QDs	T-CuInS_2_ QDs	No	Red	∆F/F_min_	0.72	[301]
	FRET (GE)	Cys-FS	Yes	Cyan/yellow	∆R/R_min_	0.42 ^b^	[251]
	FRET (Synthetic)	TP-Ratio-Cys	No	Blue/yellow	∆R/R_min_	36	[325]
		Probe 1	No	Blue/green		50	[326]
Met	FRET (GE)	FLIPM	Yes	Cyan/yellow	∆R/R_min_	0.42 ^b^	[255]
		YFP-MetQ-R189CouA		Blue/yellow		1.7	[324]
GABA	GE	iGABASnFR	Yes	Green	∆F/F_min_	4.5	[298]
	Hybrid	Pf622.V278C-JF585	No	Red	∆F/F_min_	~0.7	[298]
		GABA-Snifit	Yes	Green/far red	∆R/R_min_	0.8	[328]
Lys	FRET (GE)	ECFP-cpLAO-BP-Citrine	Yes	Cyan/yellow	∆R/R_min_	~0.83	[318]
		FLIPK				~0.26 ^b,c^	[254]
Arg	FRET (GE)	QBP/Citrine/ECFP	Yes	Cyan/yellow	∆R/R_min_	~0.25	[320]
		FLIP-cpArgT194	No			0.54	[317]
		cpFLIPR				0.35	[154]
Gly	FRET (GE)	GlyFS	No	Cyan/yellow	∆R/R_min_	0.28	[316]
Thr	QDs	T-CuInS_2_ QDs	No	Red	∆F/F_min_	0.37	[301]
Trp	FRET (GE)	FLIPW-CTYT	No	Cyan/yellow	∆R/R_min_	0.35	[321]
His	Synthetic	CAQA	No	Blue	∆F/F_min_	~18 ^c^	[289]
		NPC		Green	∆F/F_min_	6	[290]
		NCH-Cu^2+^		Green		10 ^c^	[291]
	GE	FHisJ	Yes	Yellow	∆F/F_min_	5.2	[299]
	QDs	T-CuInS_2_ QDs	No	Red	∆F/F_min_	0.46	[301]
		Y-CDs		Yellow		4.5	[302]
	FRET	FLIP-cpHisJ194	No	Cyan/yellow	∆R/R_min_	0.63	[317]
Ile	FRET	FLIP-cpLivJ261	Yes	Cyan/yellow	∆R/R_min_	0.25	[317]
		GEII	Yes			0.44 ^b^	[252]
		OLIVe	No			1.05	[322]
Leu	FRET	FLIP-cpLivJ261	Yes	Cyan/yellow	∆R/R_min_	0.25	[317]
		FLIP-Leu				~0.7 ^b^	[253]
		OLIVe	No			1.05	[322]
Val	FRET	FLIP-cpLivJ261	No	Cyan/yellow	∆R/R_min_	0.25	[317]
		OLIVe				~0.9	[322]

^a^ For sensors with multiple variants, the maximum response is reported. ^b^ These are sensors we find concerning due to an apparent lack of internal consistency in the characterization data. ^c^ Response was not explicitly reported or easily calculatable from an equation and was consequently estimated based on the provided data. ^d^ Response estimated using a non-zero minimum concentration of ligand in the linear range. ^e^ Two fluorescent species with their own responses to cysteine. ^f^ Response calculated with maximum concentration for linear range, which is below the maximum tested concentration.

## Abbreviations

AMPA	α-amino-3-hydroxy-5-methyl-4-isoxazoleproprionic acid
Asp	Aspartic acid
BBB	Blood–brain barrier
BCAAs	Branched-chain amino acids
CNS	Central nervous system
EAAT1	Excitatory amino acid transporter 1
FP	Fluorescent protein
FRET	Förster Resonance Energy Transfer
GABA	γ-Aminobutyric acid
GPCR	G protein-coupled receptors
GLAST-1	Glutamate aspartate transporter 1
GFP	Green fluorescent protein
mGluR5	Metabotropic glutamate receptor 5
MWCNT	Multiwalled carbon nanotubes
NMDA	*N*-methyl-d-aspartate
NIR	Near-infrared
NO_x_	Nitric oxide
PNS	Peripheral nervous system
PBP	Periplasmic binding protein
PA	Pipecolic acid
QD	Quantum dot
5-HT	Serotonin
SWCNT	Single-walled carbon nanotubes
Snifit	SNAP tag-based indicator proteins with a fluorescent intramolecular tether
VGAT	Vesicular GABA transporter
